# Curcumin Blocks High Glucose-Induced Podocyte Injury via RIPK3-Dependent Pathway

**DOI:** 10.3389/fcell.2022.800574

**Published:** 2022-05-30

**Authors:** Hyunsoo Chung, Seong-Woo Lee, Miri Hyun, So Young Kim, Hyeon Gyu Cho, Eun Soo Lee, Jeong Suk Kang, Choon Hee Chung, Eun Young Lee

**Affiliations:** ^1^ College of Medicine, Soonchunhyang University, Cheonan, South Korea; ^2^ Department of Internal Medicine, Soonchunhyang University Cheonan Hospital, Cheonan, South Korea; ^3^ BK21 Four Project, College of Medicine, Soonchunhyang University, Cheonan, South Korea; ^4^ Department of Internal Medicine, Yonsei University Wonju College of Medicine, Wonju, South Korea; ^5^ Institution of Genetic Cohort, Yonsei University Wonju College of Medicine, Wonju, South Korea; ^6^ Institute of Tissue Regeneration, College of Medicine, Soonchunhyang University, Cheonan, South Korea

**Keywords:** diabetic nephropathy, curcumin, necroptosis, RIPK3, antioxidant

## Abstract

Podocyte loss is well known to play a critical role in the early progression of diabetic nephropathy. A growing number of studies are paying attention to necroptosis, a programmed form of cell necrosis as a mechanism of podocyte loss. Although necroptosis is a recently established concept, the significance of receptor interacting serine/threonine kinase 3 (*RIPK3*), a gene that encodes for the homonymous enzyme RIPK3 responsible for the progression of necroptosis, is well studied. Curcumin, a natural hydrophobic polyphenol compound responsible for the yellow color of *Curcuma longa,* has drawn attention due to its antioxidant and anti-inflammatory effects on cells prone to necroptosis. Nonetheless, effects of curcumin on high glucose-induced podocyte necroptosis have not been reported yet. Therefore, this study investigated RIPK3 expression in high glucose-treated podocytes to identify the involvement of necroptosis *via* the RIPK3 pathway and the effects of curcumin treatment on RIPK3-dependent podocytopathy in a hyperglycemic environment. The study discovered that increased reactive oxygen species (ROS) in renal podocytes induced by high glucose was improved after curcumin treatment. Curcumin treatment also significantly restored the upregulated levels of VEGF, TGF-β, and CCL2 mRNAs and the downregulated level of nephrin mRNA in cultured podocytes exposed to a high glucose environment. High glucose-induced changes in protein expression of TGF-β, nephrin, and CCL2 were considerably reverted to their original levels after curcumin treatment. Increased expression of RIPK3 in high glucose-stimulated podocytes was alleviated by curcumin treatment as well as N-acetyl cysteine (NAC, an antioxidant) or GSK′872 (a RIPK3 inhibitor). Consistent with this, the increased necroptosis-associated molecules, such as RIPK3, pRIPK3, and pMLKL, were also restored by curcumin in high glucose-treated mesangial cells. DCF-DA assay confirmed that such a result was attributed to the reduction of RIPK3 through the antioxidant effect of curcumin. Further observations of DCF-DA-sensitive intracellular ROS in NAC-treated and GSK′872-treated podocyte groups showed a reciprocal regulatory relationship between ROS and RIPK3. The treatment of curcumin and GSK′872 in podocytes incubated with high glucose protected from excessive intracellular superoxide anion production. Taken together, these results indicate that curcumin treatment can protect against high glucose-induced podocyte injuries by suppressing the abnormal expression of ROS and RIPK3. Thus, curcumin might be a potential therapeutic agent for diabetic nephropathy as an inhibitor of RIPK3.

## Introduction

Diabetic nephropathy (DN) is the major cause of mortality in patients with diabetes (Dabla, 2010). Diabetes-induced chronic hyperglycemia can cause metabolic and hemodynamic abnormalities with an increase in reactive oxygen species (ROS), leading to abnormal changes in renal podocytes and DN, whose main symptoms are increased renal albuminuria and declining renal function (Dabla, 2010; Cao and Cooper, 2011).

Podocytes play multiple roles in maintaining kidney functions (Anil Kumar et al., 2014). They also play a vital role in the pathogenesis of DN. Podocytes can maintain the size of a protein and the charge of the glomerular filtration barrier, which forms a filtration unit of the kidney (Anil Kumar et al., 2014). In addition, podocytes are involved in the maintenance of capillary loop shape, the maintenance and synthesis of the GBM (glomerular basement membrane), and the production of VEGF (vascular endothelial growth factor) (Wolf et al., 2005; Jefferson et al., 2011). A decrease in the number of glomerular podocytes is positively related to the emergence of DN in diabetic patients. It is also known to play a significant role in the progression of DN (Susztak et al., 2006).

Necroptosis is a nonapoptotic form of programmed cell death with necrotic morphology. It has been demonstrated that necroptosis plays a significant role in various pathological processes, including acute renal injury (Xu et al., 2019). Since receptor-interacting protein kinase 3 (RIPK3) is a key molecule for the necroptosis pathway, the degree of necroptosis execution could be quantified with an increased level of RIPK3 (Xu et al., 2019). Additionally, recent studies have shown that an increase of ROS induced by RIPK3 elevation could initiate necroptosis in the pathophysiological process of acute renal injury (Priante et al., 2019). Hyperglycemic conditions could activate various pathways that provoke cell death. One of the major impacts of a high glucose environment caused by diabetes is necroptosis in renal podocytes (LaRocca et al., 2016; Volpe et al., 2018).

Curcumin, a natural hydrophobic polyphenol compound responsible for the yellow color in *Curcuma longa*, has gathered attention from various research fields due to its natural antioxidant, anticancer, anti-inflammatory, antiangiogenic, and antiapoptotic effects (Gururaj et al., 2002; Majithiya and Balaraman, 2005; Koeberle et al., 2009; Perrone et al., 2015; Zhang et al., 2020). The effect of curcumin on high glucose-induced podocyte injury has been discussed in few studies, mainly focusing on curcumin’s antioxidant and anti-inflammatory properties (Kanitkar et al., 2008; Meng et al., 2013; Den Hartogh et al., 2019). Curcumin has recently been reported to have a protective effect on hepatocyte or neuronal cells prone to necroptosis (Dai et al., 2013; Lu et al., 2016). There have been few studies using animal models to show improved renal function after curcumin treatment (Sharma et al., 2006; Kim et al., 2016). However, the role of curcumin in high glucose-induced podocyte necroptosis is to be clarified yet. Therefore, the objective of this study was to investigate the effect of curcumin treatment on high glucose-induced podocyte injury caused by oxidative stress or inflammatory responses.

## Materials and Methods

### Chemicals

Dulbecco’s modified Eagle’s medium (DMEM), fetal bovine serum (FBS), and phosphate buffered saline (PBS) were purchased from Gibco (Grand Island, NY, United States). Trypsin/EDTA (#25-053-cl) and penicillin/streptomycin (#30-002-cl) were purchased from Corning (Corning, NY, United States). TNF-α (#AFL410) was purchased from R&D Systems (Minneapolis, Minnesota, United States). Curcumin (diferuloylmethane), DHZ [1,7-bis(4-hydroxy-3-methoxyphenyl)-1,6-heptadiene-3,5-dione], and reagents were purchased from Sigma (Paterson, NJ, United States). Curcumin dissolved in DMSO obtaining a concentration of 100 μg/ml was stored at −70°C. If necessary, it was diluted to various concentrations either in the cell culture medium or PBS for its use.

### Cell Culture and Glucose Treatment

Conditionally immortalized mouse podocytes were kindly provided by Dr. Peter Mundel and cultured as described previously (Kang et al., 2019; Lee et al., 2019). Cell proliferation was retained in DMEM supplemented with 10% FBS with 10 U/mL IFN-γ and 1% penicillin/streptomycin at 33°C. Cell differentiation was induced in culture media without IFN-γ at 37°C for 14 days to develop podocyte foot processes similar to *in vivo*. Differentiated mouse podocytes were then challenged with either normal (5.6 mM) or high (30 mM) glucose medium for 24 h. For high glucose treatment, glucose powder [D-(+)-Glucose, #G7528, Sigma] was dissolved to obtain a 3 M stock. It was then used to treat the immortalized mouse podocytes. Mouse mesangial (MES-13) was purchased from the American Type Culture Collection (ATCC, Manassas, VA). MES-13 cells were cultured in DMEM containing 5.5 mM glucose, 1% antibiotics, and 10% FBS. The cultured cells were starved for 24 h and then stimulated with high glucose with or without 1 μM of curcumin and 1 μM of DHZ for 24 h.

### Assessment of Cell Viability

To determine the toxicity of curcumin, the MTT [3-(4,5-dimethylthiazol-2-yl)-2,5-diphenyltetrazolium bromide] assay was performed. Using trypsin/EDTA, the cultured cells were detached from the culture plate and the cell suspension solution was put into a 96-well culture plate after measuring the optimum cell concentration (1 × 10^5^/ml) *via* a hemocytometer. After the plate was incubated at 37°C for 24 h, different concentrations of curcumin were added to the serum-free culture medium and the plate was cultured for another 24 h. After removing the medium, 100 μL of the MTT solution was added to each well. Following incubation for 2 h, the solution was carefully removed and 100 μL DMSO was added to each well. The cells were extracted to measure the absorbance at a wavelength of 540 nm using a microplate reader.

### Measurement of Intracellular ROS

To determine the effect of curcumin on intracellular ROS expression, after washing the 2-week incubated podocytes twice with PBS, 5M of oxidation-sensitive 2′-7′ dichlorofluorescein diacetate (DCF-DA, Invitrogen, Grand Island, NY, United States) fluorescent probe was added followed by incubation at 37°C for 10 min. To examine the effect of GSK′872 or N-acetyl-L-cysteine on ROS expression in the high glucose-treated podocytes, 10 µM stock of DCF-DA was dissolved in the culture media and added to cells. After incubating at 37°C for 30 min, excess dye was washed away. DCF-DA sensitive intracellular ROS (reactive oxygen species) was then observed and quantified using a Carl Zeiss LSM 710 confocal microscope.

### Superoxide Anion Assay

To measure the superoxide anion, one of the ROS types, the superoxide anion assay kit (# CS1000, Sigma) was used. The mouse podocytes were transferred to a 96-well plate (3 × 10^5^ cells/well) on the 10^th^ day after the start of the differentiation, and 2 days later, they were starved for 24 h. After treating the cells with GSK′872, curcumin or NAC in high glucose condition for 16 h, the medium was replaced with the assay medium (A5980) included in the superoxide assay kit and incubated at 37°C for 10 min. Then, luminol solution (L5043), xanthine oxidase from milk (X4878), superoxide dismutase (S6696), and enhancer solution (E4281) were added to the assay buffer (A5980) according to the conditions, and then reacted at 37°C for 30 min. The luminescence was measured using GloMax^®^ Discover Microplate Reader (GM3000, Promega).

### Enzyme-Linked Immunosorbent Assay

ELISA was performed using ELISA kits purchased from R&D Systems (Minneapolis, MN, United States) to analyze the supernatant CCL2 (C-C motif chemokine ligand 2) from the podocytes. After placing 100 μL of the sample into the well of a 95-well microplate, it reacted at room temperature for a given time. After washing the sample four times with a washing buffer, 200 μL conjugate was added to each well and reacted for 2 h. After washing with the washing buffer four times, 200 μL of the substrate solution was added and reacted without light for 20 min. After adding 50 μL of the stop solution to each well, the absorbance at 450 nm wavelength was measured using a microplate reader.

### Quantitative Real-Time PCR

The total RNA was extracted using TRIzol purchased from Invitrogen (Carlsbad, CA, United States) following the manufacturer’s protocol. cDNA was synthesized from 0.5 to 1 μg of the total RNA using a ReverTraAce^®^ qPCR RT Master Mix (TOYOBO, Japan) according to the manufacturer’s protocol. For PCR, 10 ng of cDNA, SYBR PCR master mix plus (TOYOBA, Osaka, Japan), and primer were mixed to make a total volume of 20 μL. PCR for CCL2, TGF (transforming growth factor)-β, and VEGF (vascular endothelial growth factor) used the gene-specific primers as follows: β-actin, 5′-cca tga aga tca aga tca ttg ctc c-3’ (forward), and 5′-tc ttg t atc cac atc tgc t-3’ (reverse); CCL2, 5′-ctg gat cg aac caa atg ag-3’ (forward) and 5′-cgg gtc aac ttc aca ttc aa-3’ (reverse); TGF-β, 5′-agc ccg aag cgg act act at-3’ (forward), and 5′-ct tgt gag atg tct ttg gtt ttc-3’ (reverse); and VEGF, 5′-gta cat ctt caa gcc gtc ctg tgt-3’ (forward) and 5′-tcc gca tga tct gca tgg tg-3’ (reverse). Real-time PCR was performed using CFX-96 (BIO-RAD). The condition of PCR used in this study was: 40 repeated cycles of 30 s at 95°C, 5 s at 95°C, 10 s at 58°C, and 15 s at 72°C. Expression analysis was performed using the ΔΔC_T_ method. Expression levels of all genes were normalized against the expression of β-actin in the same sample (Schefe et al., 2006).

### Western Blot

Protein expression levels of RIPK3, TGF-β, and nephrin were measured by Western blot analysis using specific antibodies. After 24 h of treatment with different concentrations of glucose and curcumin, the media were removed and the plates were washed with cold PBS three times. These plates were stood upright and refrigerated at 4°C for 10 min to remove the remaining PBS/media with a micropipette. Then 90 μL of PRO-PREPTM protein extraction solution (iNtRON biotechnology, Seoul, Korea) was added to collect the podocytes and to extract the proteins. The protein extract was quantified by Lowry assay (BIO-RAD). SDS-PAGE was performed after adding the Laemmli buffer. The proteins were then transferred to the nitrocellulose membranes. The membranes were blocked with 1xPBS/T containing 5% skim milk (Merck, Rahway, NJ, United States) for an hour and then incubated with a primary antibody at 4°C overnight. The primary antibodies of β-actin (#sc-47778, 1:5,000, Santa Cruz), RIPK3 (#AHP1797, 1:1,000, Synaptic Systems), pRIPK3 (#ab195117, 1:1,000, Abcam), pMLKL (#ab196436, 1:1,000, Abcam), TGF-β (#sc-146, 1:500, Santa Cruz), and nephrin (#GP-N2, 1:500, Progen) were used.

The membranes were washed with 1xPBS/T three times for 10 min each and reacted with secondary antibodies, either mouses-HRP (#W4028, 1:5,000, Promega), rabbit-HRP (#31460, 1:2,000, Invitrogen), or guineapig-HRP (#sc-2438, Santa Cruz) for 3.5 h at 4°C. After washing again with 1xPBS/T three times for 10 min each, the membranes were developed with an ECL kit. Protein band images were obtained using Chemidoc (BIO-RAD). Intensities of obtained protein bands were quantified with ChemiDocTM XRS+ (Bio-RAD) imaging system using a Luminata Forte enhanced chemiluminescence solution (Millipore).

To further clarify the mechanism of curcumin’s protective effect on RIPK3 production, Western blot analysis of RIPK3 and β-actin protein expressions was proceeded by abiding the same procedure as previously described except that the cell plate treatment was different. Cell plates were incubated in a serum-free condition for 24 h. They were then incubated for another 24 h with 0.2% FBS media containing normal or high glucose and 10 μM of GSK′872 (RIPK3 inhibitor, #5. 30389. 0001, EMD Millipore) or 50 μM of N-acetyl-L-cysteine (NAC) (antioxidant, #a7250, Sigma).

### Statistical Analysis

Statistical comparisons were performed with Student’s t-test. All analyses were performed with SPSS software. Experimental values are presented as mean ± SD using data obtained from at least three independent experiments. *p*-value < 0.05 was considered statistically significant.

## Results

### MTT Assay

To establish the experimental conditions in which cellular toxicity would be minimized, the renal podocytes in a 96-well plate were treated with 0, 50, 100, 150, and 200 μM of curcumin for 24 h for the MTT assay. Curcumin concentration less than 50 uM did not show cytotoxicity, while cell viability was reduced to 77% at 100 uM and to 30% at 150 uM and 200 uM compared to the normal control (Supplementary Figure S1). Based on these results, 50 uM curcumin was used in this study.

### Curcumin Treatment Alleviates ROS Generation in High Glucose-Treated Podocytes

**FIGURE 1:**
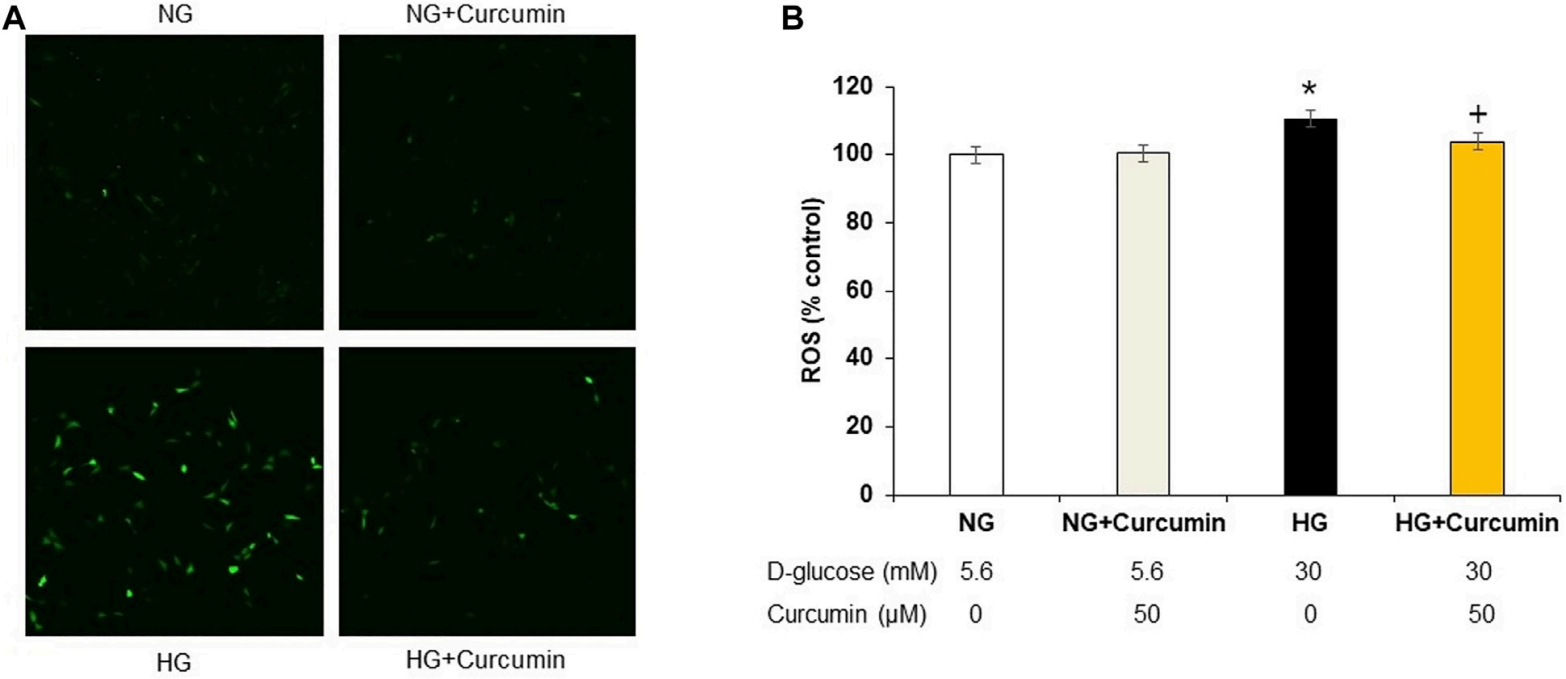
Curcumin decreases ROS expression in high glucose-treated podocytes. DCF-DA-sensitive intracellular ROS was measured after incubating podocytes with NG (5.6 mM D-glucose), NG + Curcumin (5.6 mM D-glucose + curcumin), HG (30 mM D-glucose), and HG + Curcumin (30 mM D-glucose + curcumin) for 24 h. HG-induced intracellular ROS was markedly reduced by curcumin treatment **(A,B)**. 2′-7′ DCF-DA, dichlorofluorescein diacetate DCF-DA; NG, normal glucose; HG, high glucose. *, *p* < 0.05 *versus* NG; + *p* < 0.05 *versus* HG.

Increased DCF-DA sensitive intracellular ROS generation under 30 mM high glucose condition was observed by using a confocal microscope (Figure 1A) and quantified by using a fluorometer (Figure 1B). The high glucose-treated podocytes showed significantly increased ROS expression. However, the curcumin treatment significantly reduced such changes.

### Curcumin Treatment Decreases CCL2 Protein and mRNA Levels in High Glucose-Treated Podocytes

**FIGURE 2:**
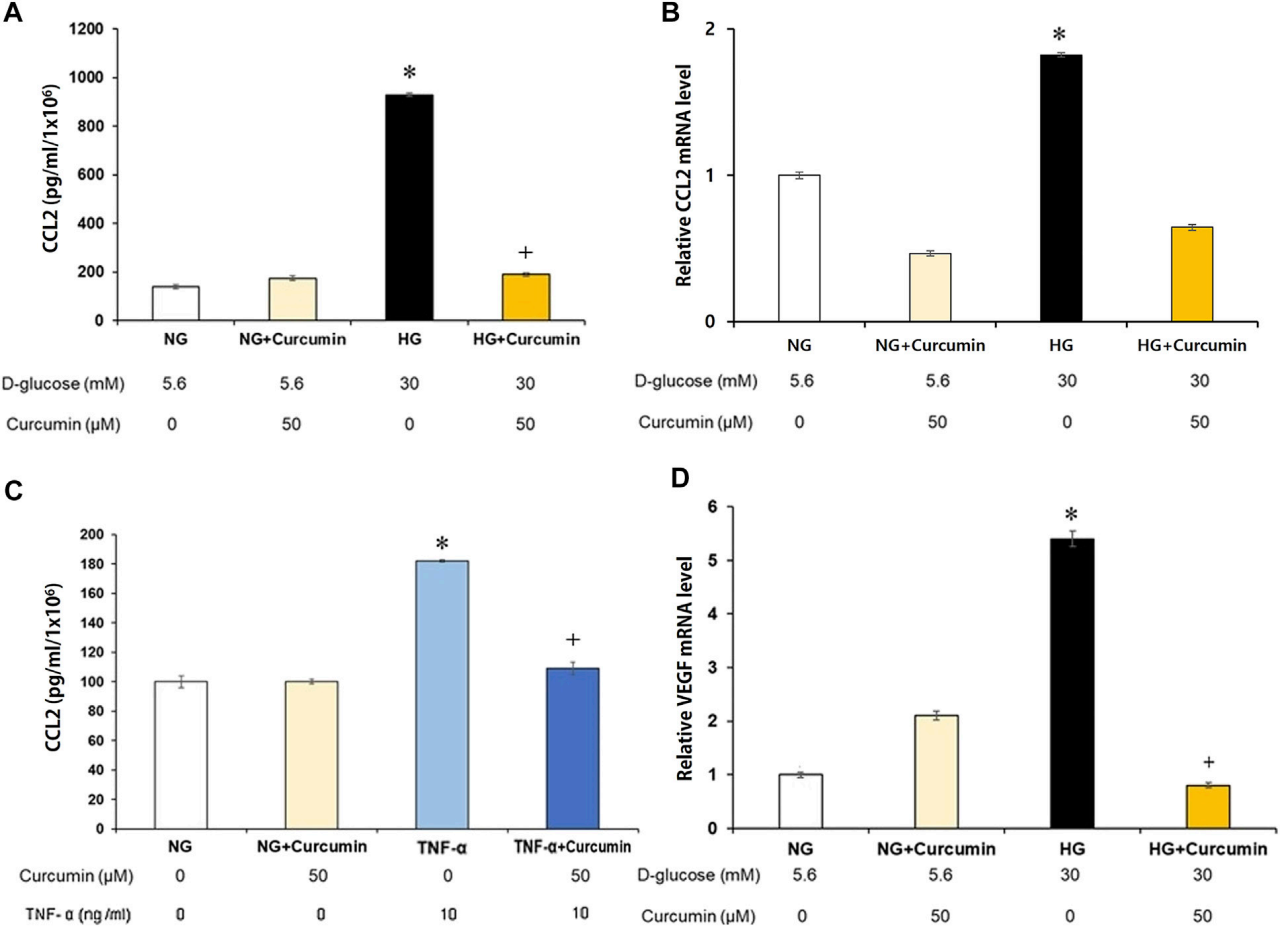
Curcumin reduces high glucose-induced CCL2 and VEGF expression in renal podocytes. Supernatant CCL2 was measured by ELISA **(A,C)**, and podocyte CCL2 mRNA was measured by real-time PCR **(B)**. High glucose-induced CCL2 protein **(A)** and mRNA expression **(B)** were markedly improved after curcumin treatment. Podocytes treated with 10 ng/ml of TNF-α for 24 h increased the CCL2 protein expression. Curcumin treatment significantly reduced TNF-α-induced CCL2 protein in the podocytes **(C)**. High glucose-induced VEGF mRNA expression was significantly reduced by curcumin **(D)**. Vascular endothelial growth factor, VEGF; C-C motif chemokine ligand 2, CCL2; NG, normal glucose; HG, high glucose. *, *p* < 0.05 *versus* NG; **, *p* < 0.001 *versus* NG; +, *p* < 0.05 *versus* HG or TNF-α.

Supernatant CCL2 protein secreted by the high glucose-treated podocytes was analyzed. The results showed a 10-fold increase of the CCL2 protein expression compared to that of the normal glucose-treated podocyte group. Curcumin treatment significantly attenuated the elevated CCL2 expression to a certain extent, although not to the normal level (Figure 2A). The CCL2 mRNA expression was similarly increased more than 1.8 times greater in response to high-glucose (30 mM) stimulation. However, it was remarkably reduced back to the normal level after the curcumin treatment (Figure 2B). When the podocytes were stimulated with the inflammatory cytokine TNF-α (tumor necrosis factor α), they showed 1.8-fold increase in the supernatant CCL2 protein expression. However, the supernatant CCL2 protein expression was significantly recovered to normal level after the treatment with curcumin (Figure 2C).

### Recovery of the VEGF Expression Level After Curcumin Treatment

The high-glucose-stimulated podocytes showed a 5.3-fold increase of the VEGF mRNA expression than the podocytes cultured in a normal glucose (5.6 mM) environment. However, the VEGF mRNA expression was markedly decreased to a normal level after curcumin treatment (Figure 2D).

### Curcumin Treatment Restores High Glucose-Mediated Reduction in the Expression of Nephrin

**FIGURE 3:**
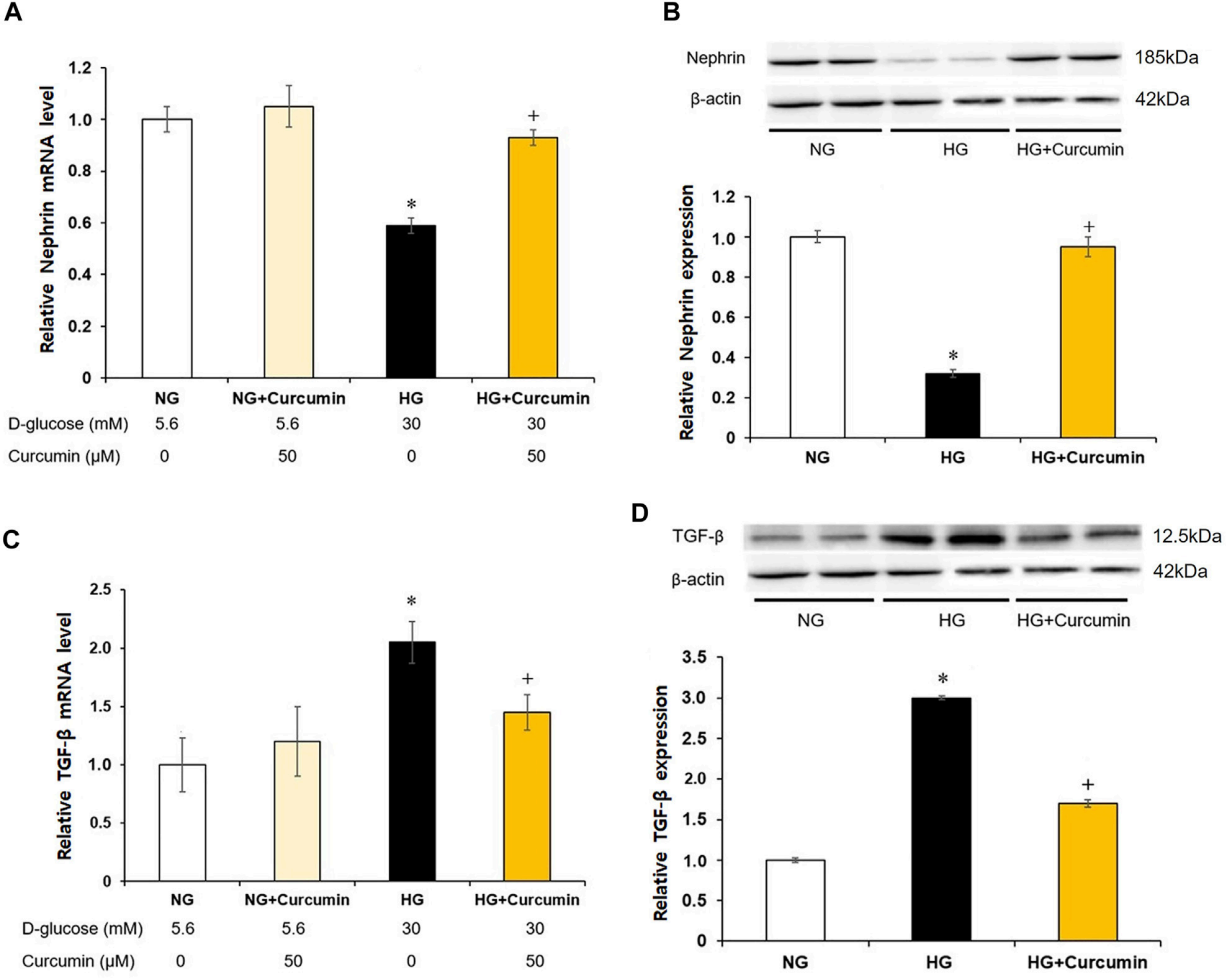
Curcumin restores decreased nephrin and increases TGF-β expression in high glucose-treated podocytes. Podocytes were treated with high glucose with or without 50 μM of curcumin for 24 h. Decreased nephrin expression at mRNA **(A)** and protein **(B)** levels in high-glucose environment was significantly restored after curcumin treatment. High glucose-induced increases of TGF-β expression at mRNA **(C)** and protein **(D)** levels were markedly improved after curcumin treatment. NG, normal glucose; HG, high glucose. *, *p* < 0.05 *versus* NG; +, *p* < 0.05 *versus* HG.

The podocytes stimulated with a high glucose showed nearly 0.4-fold decrease in the nephrin mRNA expression (Figure 3A) and approximately 0.6 times reduction in the nephrin protein expression (Figure 3B) than the differentiated podocytes treated with normal glucose. However, these changes were all significantly recovered by the curcumin treatment.

### Reduced Expression of TGF-β at Protein and mRNA Levels After Curcumin Treatment

Compared with differentiated podocytes cultured in normal glucose, the podocytes stimulated with high glucose showed a 2-fold increase in TGF-β mRNA expression (Figure 3C) and a 3-fold increase in TGF-β protein expression (Figure 3D). However, these increases of the TGF-β mRNA and protein expression levels were significantly decreased after the curcumin treatment.

### Increased RIPK3 Expression Is Recovered by Curcumin and Antioxidant NAC Treatment

**FIGURE 4:**
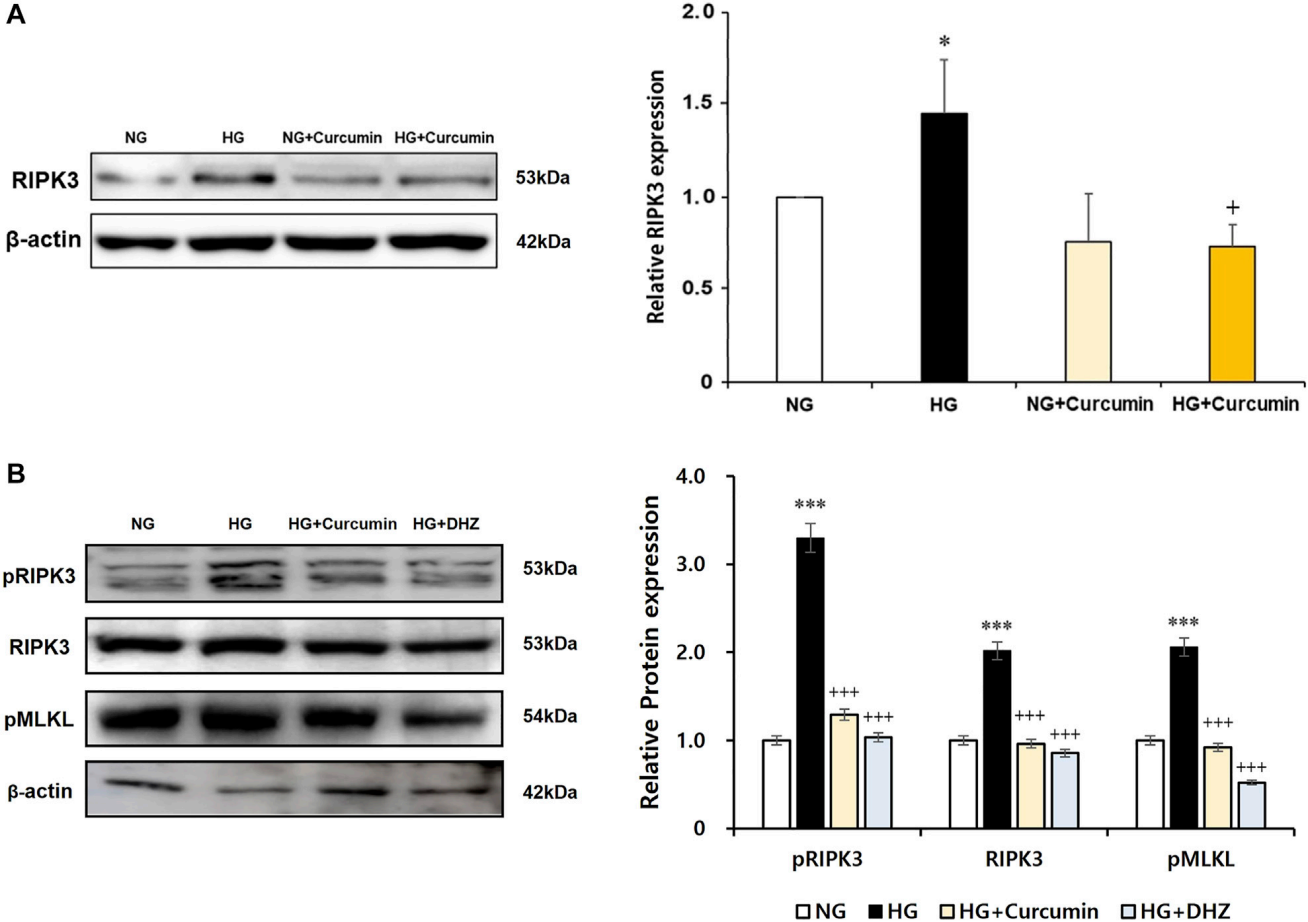
Curcumin works as an inhibitor of RIPK3 to protect renal podocytes and MES-13 cells from high glucose-induced injury. High glucose-induced RIPK3 overexpression in podocytes was significantly reduced by curcumin treatment **(A)**. Representative immunoblot for RIPK3, pRIPK3, and pMLKL by Western blot in mouse mesangial (MES-13) cells treated with high glucose with or without 1 μM of curcumin and 1 μM of DHZ for 24 h **(B)**. NG, normal glucose; HG, high glucose. *, *p* < 0.05 *versus* NG; +, *p* < 0.05 *versus* HG.

**FIGURE 5:**
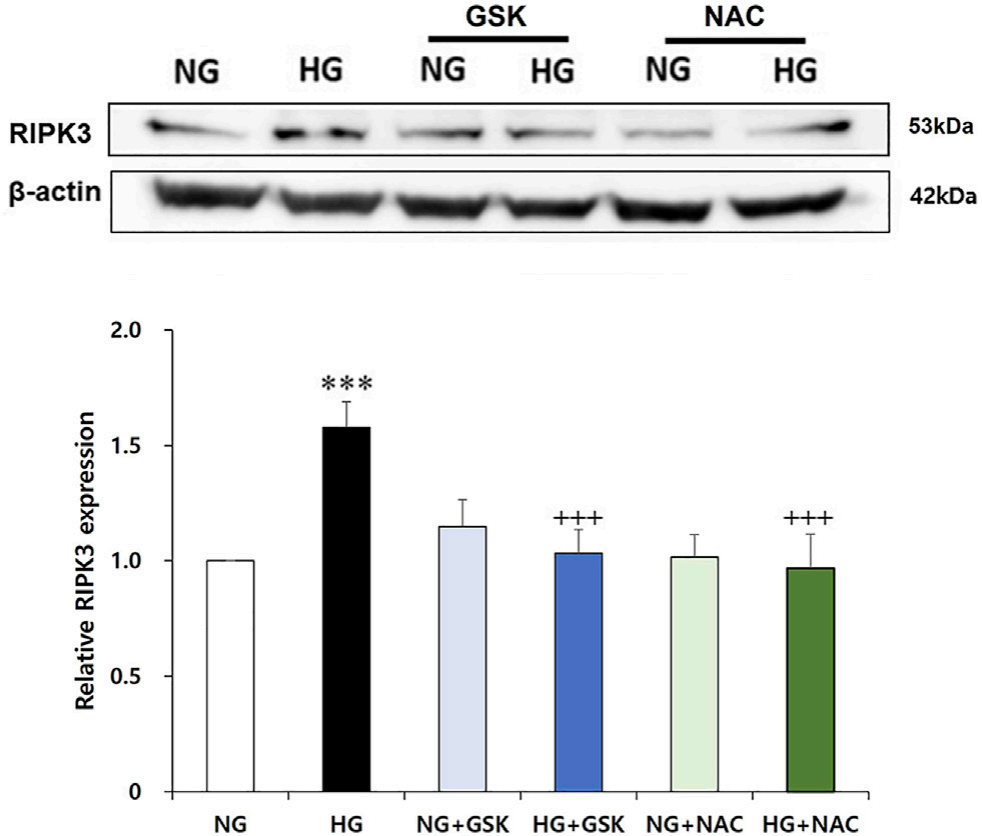
GSK′872 and N-acetyl cysteine equally suppress RIPK3 expressions in high glucose-treated renal podocytes. A histogram shows RIPK3 expression in HG, GSK′872, or antioxidant-treated podocytes. Subsequent to 24-h starvation using serum-free media, each cell plate of podocytes was incubated with HG (30 mM), GSK′872 (RIPK3 inhibitor, 10 μM), or an antioxidant (NAC, 50 μM) for another 24 h. Plates were then suctioned and washed three times with PBS. Podocytes were harvested by adding 90 μL of PRO-PREP™ solution to each cell plate to perform Western blot analysis. The expression of RIPK3 was significantly lower in both GSK′872-treated and NAC-treated cell plates, which did not show any statistically significant difference from the negative control group. NG, normal glucose; HG, high glucose; GSK, GSK′872; NAC, N-acetyl cysteine. ***, *p* < 0.001 *versus* NG; +++, *p* < 0.001 *versus* HG.

As a result of the culturing podocytes exposed to high glucose for 24 h, the expression of RIPK3 was increased approximately 1.5-fold compared to that in the podocytes treated with normal glucose. When the high glucose-treated podocytes were cotreated with curcumin, however, the increased RIPK3 expression was significantly decreased similar to that in the normal control (Figure 4A). Mouse MES-13 cells, another renal cell line, were used to verify the increased expression of RIPK3 by high glucose. As shown in Figure 4B, RIPK3 expression was increased in the high glucose-treated mesangial cells but decreased by the treatment of curcumin and DHZ (dehydrozingerone), a structural analog of curcumin. In addition, it was confirmed that the expression of pRIPK3 and pMLKL increased by the high glucose was also decreased by curcumin and DHZ treatment, like RIPK3. In addition, the high glucose-mediated RIPK3 overexpression was restored by an antioxidant NAC treatment as shown in the result for RIPK3 inhibitor GSK′872 (Figure 5). These results suggest that curcumin might protect podocyte injury by regulating the expression of RIPK3 increased by hyperglycemia in a diabetic environment.

### Reciprocal Regulatory Relationship Between ROS and RIPK3

**FIGURE 6:**
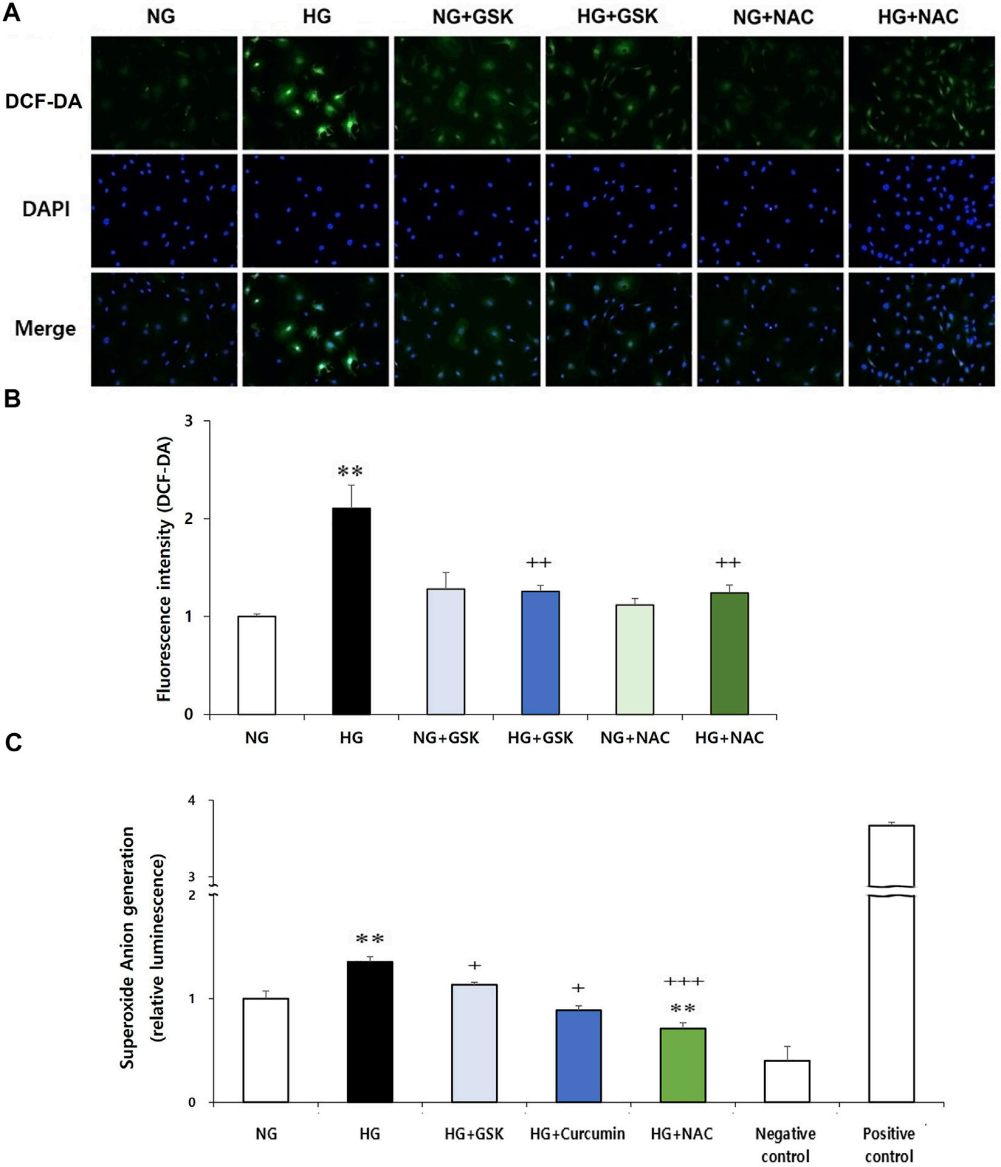
GSK′872 or NAC treatment reduces intracellular ROS expression in high glucose-treated podocytes. DCF-DA-sensitive intracellular ROS was observed **(A)** and quantified **(B)** after treating the podocytes under six different conditions for 24 h: NG (5.6 mM D-glucose), HG (30 mM D-glucose), NG + GSK (5.6 mM D-glucose + 10 µM GSK′872), HG + GSK (30 mM D-glucose + 10 µM GSK′872), NG + NAC (5.6 mM D-glucose + 50 μM N-acetyl cysteine), and HG + NAC (30 mM D-glucose + 50 μM N-acetyl cysteine). In GSK- or NAC-treated groups, HG-induced intracellular ROS expression was significantly reduced **(A,B)**. NG, normal glucose; HG, high glucose; ROS, reactive oxygen species; GSK, GSK′872. **, *p* < 0.01 *versus* NG; ++, *p* < 0.01 *versus* HG. Superoxide anion was measured using a luminometer. After starvation for 24 h, drug treatment was performed for 16 h **(C)**. High glucose-induced superoxide anion production in podocytes was significantly reduced by curcumin, GSK′872, and NAC. Negative control, assay buffer + xanthine oxidase + SOD; positive control, assay buffer + xanthine oxidase. **, *p* < 0.01 *versus* NG; +, *p* < 0.05, ++, *p* < 0.01 *versus* HG.

Next, to investigate the interaction between RIPK3 and ROS in podocyte injury, effects of RIPK3 inhibitor GSK′872 and NAC on ROS generation in the high glucose-treated podocytes were investigated. As shown in Figures 6A,B, while the intracellular ROS production was increased in the high glucose-treated podocytes compared to that in the control, ROS levels were decreased by the RIPK3 inhibitor GSK′872. Similarly, ROS was decreased by the NAC treatment. In addition, the podocytes stimulated with high glucose had higher level of intracellular superoxide anion production compared to the cells exposed to normal glucose. Treatment of curcumin, GSK′872, and NAC in podocytes exposed to high glucose can lower the levels of intracellular superoxide anion than in the podocytes exposed to high glucose (Figure 6C).

## Discussion

Previous studies about curcumin’s protective effect on the renal podocytes were mainly focused on curcumin’s anti-inflammatory and antioxidant effects and mechanisms (Sharma et al., 2006; Den Hartogh et al., 2019). Although some animal experiments have confirmed curcumin’s protective effect on podocyte apoptosis or general renal damage (Sharma et al., 2006; Kim et al., 2016), the effect and mechanism of curcumin on podocytes necroptosis have not been clearly demonstrated yet. Thus, this study aimed to investigate curcumin’s effects on high glucose-induced necroptosis of the renal podocytes by observing the alterations in RIPK3 level.

The major finding of this study was that high glucose exposure increased the RIPK3 expression in the renal podocytes and that this change was restored to normal levels by curcumin treatment. Western blot analysis of the RIPK3 expression after treatment with GSK′872 or NAC confirmed that curcumin functioned as an inhibitor of RIPK3 and an antioxidant. Our study is the first to demonstrate the potential role of curcumin as an inhibitor of RIPK3 in the renal podocytes. High glucose-induced RIPK3 overexpression was significantly reduced after curcumin treatment. Since the previous studies have shown that the RIPK1/RIPK3 pathway is a major regulator of podocyte necroptosis (Xu et al., 2019), the potential application of curcumin to minimize hyperglycemic damage to the kidney is compelling.

Our study showed that an increased intracellular ROS expression induced by high glucose exposure was recovered by the curcumin treatment, similar to other previous experiments confirming curcumin’s antioxidant property (Balasubramanyam et al., 2003; Barzegar and Moosavi-Movahedi, 2011; Trujillo et al., 2013). ROS are the major components that can regulate necroptotic signaling. High glucose-induced intracellular ROS generation is the cause of podocytes death and loss in DN. Various types of ROS can be expressed in diabetic environment. NOX-derived ROS is known to be the most important one both numerically and functionally in the kidney cells (Jha et al., 2016). The recovery of intracellular ROS overexpression observed in this study consolidates curcumin’s ability to suppress intracellular ROS generation in the renal podocytes prone to a high glucose environment.

CCL2 is an important inflammatory chemokine that regulates the recruitment and activation of monocytes and macrophages (Lee et al., 2009). CCL2 is also one of the downstream cytokines produced by oxidative stress in diabetic conditions (Jha et al., 2016). Under diabetic conditions, CCL2 expression is upregulated in various kidney cell lines, leading to proliferation of mesangial cells, glomerulosclerosis, and kidney fibrosis (Lee et al., 2009). The mRNA expression of TNF-α, a potent cytokine, is believed to play a major role in diabetic nephropathy. It is increased in diabetic condition. As a consequence, inflammatory responses such as local generation of ROS and podocytes’ CCL2 production can occur in the kidney (Lee et al., 2009). We showed that the CCL2 level was significantly decreased after the curcumin treatment in both the HG + curcumin and TNF-α + curcumin groups, suggesting that curcumin could prevent the development of an inflammatory response. In this study, as curcumin treatment reduced ROS and CCL2 expression levels, it was confirmed that curcumin had an anti-inflammatory effect on the podocytes in diabetic state. As previously mentioned, the activation of RIPK3 resulted in oxidative stress due to the progression of necroptosis and an elevation of CCL2 and TNF-α might appear. The decrease in the CCL2 expression after the curcumin treatment is likely because of curcumin’s activity as an antioxidant and an inhibitor of RIPK3, leading to the reduced ROS expression and an anti-inflammatory response.

This study also revealed that curcumin treatment could suppress mRNA and protein overexpression levels of VEGF, a critical factor for angiogenesis, in the high glucose-induced podocytes. VEGF produced by the podocytes plays an important role in maintaining glomerular endothelial cells and the glomerular filtration barrier. The level of VEGF must be precisely controlled as both lower and higher than normal levels of VEGF may cause renal problems. Excessive VEGF level can lead to pathological microangiopathy, subsequently leading to neovascularization (Gil et al., 2021). Curcumin’s role in inhibiting the VEGF expression is supported by the previous clinical trials and *in vivo* studies (Sawatpanich et al., 2010; Wang and Chen, 2019). The decrease in the VEGF level followed by curcumin and GSK′872 in this study reflected curcumin’s role as an inhibitor of RIPK3 to suppress the vascular permeability promoted in necroptosis. As the increase of renal VEGF expression in DM patients’ glomerulus is a well-known triggering factor of diabetic nephropathy (Tufro and Veron, 2012), therapeutic use of curcumin to reduce the VEGF expression is expected to help prevent diabetic kidney disease progression. However, further studies are needed.

Observing a high glucose-induced decrease in the mRNA and protein expression of nephrin was recovered after the curcumin treatment, suggesting curcumin’s RIPK3 inhibitor activity to protect the renal podocytes against stress and inflammatory effects induced in hyperglycemic conditions. Since nephrin is a major protein secreted by the podocytes to form an integral part of primary renal function, nephrin’s reduction is closely related to increased proteinuria, expansion of GBM, and decreased slit pore density (Cooper et al., 2002). The recovered expression of nephrin after curcumin treatment in this study shows curcumin’s potential in preventing podocyte damage and maintaining its function.

Active TGF-β signaling can modify gene transcription *via* phosphorylation and translocation of Smad protein. This change can lead to podocyte apoptosis, foot effacement, and decreased VEGF production that can result in endothelial cell death (Gil et al., 2021). It has also been reported that an increased expression of CCL2 in the mesangial cells can stimulate collagen deposition, extend the mesangial matrix, and mediate collagen deposition and fibrosis in diabetic nephropathy (Lee et al., 2009; Epstein et al., 1994). As shown in Figure 3, high glucose-induced increases of the TGF-β mRNA and protein expression were significantly reduced after the curcumin treatment. As TNF-α and TGF-β coexist as downstream inflammatory cytokines in RIPK3 signaling, curcumin’s function as an inhibitor RIPK3 can reduce the fibrotic response in the kidney due to the TGF-β and CCL2 overexpression stimulated by TNF-α (Jha et al., 2016; Bertheloot et al., 2021).

As previously mentioned, this study clarified that curcumin treatment could reduce high glucose-induced ROS and RIPK3 production in the podocytes and alleviated the increase of ROS generation after the treatment with RIPK3 inhibitor GSK′872. These findings suggest that ROS generation depends on RIPK3 pathway, which is supported by a recent study investigating lyso-Gb3-induced oxidative stress *via* RIPK3-dependent pathway and implicating that external stress factors can result in ROS generation *via* RIPK3-dependent pathway (Kim et al., 2021). In addition, observing a significant recovery of the abnormally increased intracellular ROS expression followed by GSK′872 or NAC treatment confirmed the interdependence between ROS and RIPK3. Our results collectively suggested that the antioxidant property of curcumin may inhibit RIPK3 overexpression, ultimately contributing to the normalization of the ROS expression.

Existing RIPK3 inhibitors such as GSK′872 are man-made synthetic materials that can specifically prevent cell necroptosis by blocking the RIPK3 domain. Multiple studies have emphasized curcumin’s advantage over synthetic compounds, mainly due to its convenience and fewer side effects. In addition, revealing the novel properties of curcumin such as antiangiogenic effect, antiapoptotic effect, and anticancer effect is what makes curcumin a distinctive RIPK3 inhibitor (Gururaj et al., 2002; Perrone et al., 2015; Zhang et al., 2020). In the field of DM particularly, it has been confirmed that curcumin’s antioxidative and anti-inflammatory properties can ameliorate the consequences of DM without any side effects (Meng et al., 2013; Den Hartogh et al., 2019). What differentiates curcumin from the existing RIPK3 inhibitors is that curcumin not only targets necroptosis, but also has a comprehensive role in improving the damages caused by DM. Using GSK′872, an inhibitor specific for RIPK3, may be more appropriate to use when studying necroptosis alone. However, to investigate a treatment target related to the overall improvement of the disease, it seems to be more useful to confirm curcumin’s efficacy.

**FIGURE 7:**
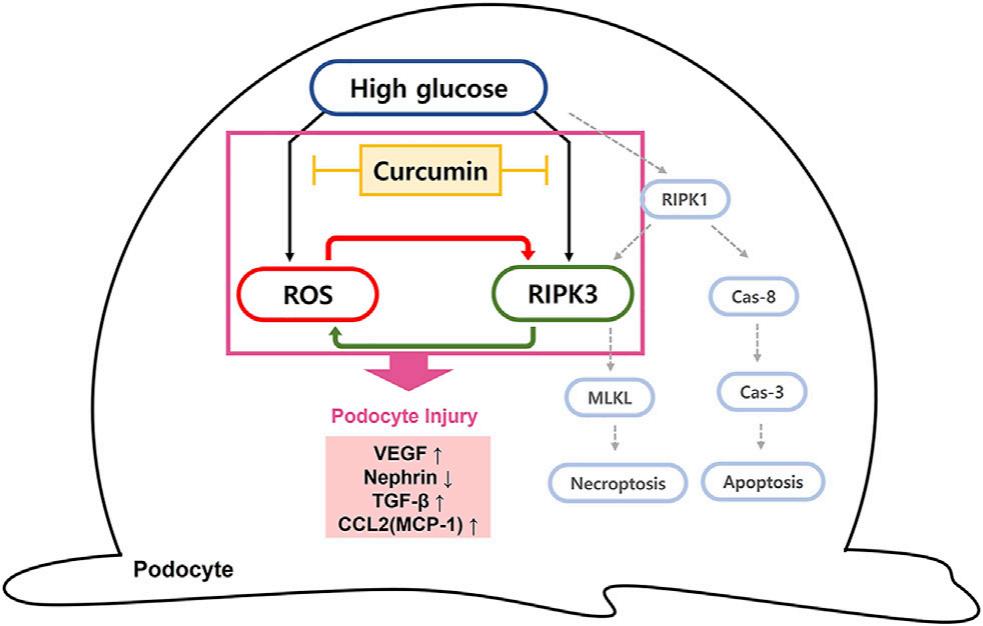
Diagram of curcumin suppressing intracellular ROS and RIPK3 expression in high glucose-treated podocytes. Schematic representation of the possible signaling pathway leading to podocytopathy by high glucose. We suggest that the elevated hyperglycemia condition can induce the intracellular ROS generation and increase RIPK3 expression, resulting in podocytopathy through inflammatory response and fibrosis. Curcumin administration protects against podocyte injury by inhibiting ROS generation and downregulating RIPK3. Due to a cross-stimulating relationship between the two, the reduced level of each further contributes to the suppression effect of curcumin on intracellular ROS and RIPK3 expression. RIPK3 inhibition prevents necroptosis of podocytes since it prevents phosphorylation and aggregation of MLKL to proceed the final stage of necroptosis. Cas-8, caspase-8; Cas-3, caspase-3; MLKL, mixed lineage kinase domain-like protein.

The ability of curcumin to regulate ROS and RIPK3 overexpression is expected to prevent podocytopathy by suspending the progression to necroptosis. A decreased ROS level is thought to reduce the incidence of necroptosis as ROS works as a stimulating factor for necroptosis. The inhibition of RIPK3 expression by curcumin can prevent the occurrence of necroptosis because without RIPK3 activation, MLKL, the terminal protein in necroptosis, cannot be phosphorylated or aggregated to proceed the final stage of necroptosis (Figure 7). In mesangial cells, high glucose-induced activation of RIPK3 and MLKL was inhibited by the treatment of curcumin and DHZ. Additional studies checking levels of necroptosis-related proteins (RIPK1, RIPK3, and MLKL) are needed to further verify the protective effect of curcumin on podocytes necroptosis.

## Conclusion

In summary, our study showed that curcumin had protective effects against oxidative stress, inflammatory response, and fibrosis in high glucose-induced podocyte injury, eventually improving podocyte function. These renoprotective effects of curcumin might be associated with its ability to inhibit high glucose-induced RIPK3 expression by inhibiting oxidative stress. Our observations suggest that curcumin might be a potential therapeutic agent to minimize the progression of podocytopathy caused by diabetes as an inhibitor of RIPK3.

## Data Availability

The raw data supporting the conclusion of this article will be made available by the authors, without undue reservation.
