# Supplementary material for: Curcumin Blocks High Glucose-Induced Podocyte Injury via RIPK3-Dependent Pathway
Source: Front Cell Dev Biol. 2022 May 30;10:800574. doi: 10.3389/fcell.2022.800574 (PMC9189280; doi:10.3389/fcell.2022.800574)
Supplement: Supplementary file 1 [file Image1.pdf]

Curcumin blocks high glucose-induced podocyte injury via RIPK3 - dependent pathway

Hyunsoo Chung[1†], Seong-Woo Lee[2,3†], Miri Hyun[2†], So Young Kim[2], Hyeon Gyu Cho[1], Eun Soo Lee[4,5], Jeong Suk Kang[2,6], Choon Hee Chung[4,5], Eun Young Lee[1,2,3,6*]

[1] College of Medicine, Soonchunhyang University, Cheonan, Korea

[2] Department of Internal Medicine, Soonchunhyang University Cheonan Hospital, Cheonan, Korea

[3] BK21 Four Project, College of Medicine, Soonchunhyang University, Cheonan, Korea

[4] Department of Internal Medicine, Yonsei University Wonju College of Medicine, Wonju, Korea

[5] Institution of Genetic Cohort, Yonsei University Wonju College of Medicine, Wonju, Korea

[6] Institute of Tissue Regeneration, College of Medicine, Soonchunhyang University, Cheonan, Korea

[†] These authors contributed equally to this work as co-first authors.

*** Correspondence:**
Eun Young Lee, MD, PhD
eylee@sch.ac.kr

Keywords: diabetic nephropathy, curcumin, necroptosis, RIPK3, antioxidant

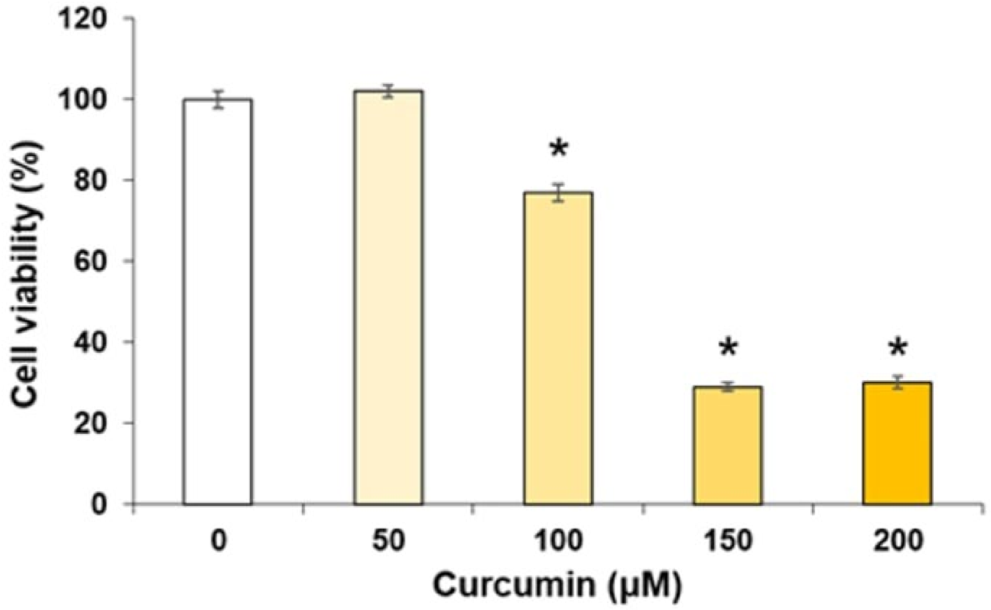

Supplementary Figure 1. Cytotoxic concentration of curcumin. Podocytes were treated with curcumin at indicated concentrations for 24 h. Cell viability was determined by MTT assay. Doses ranging from 100 to 200 µM caused decrement of cell viability. *, $p < 0.05$.
